# Machine Learning-Based Morphological Classification and Diversity Analysis of Ornamental Pumpkin Seeds

**DOI:** 10.3390/foods14091498

**Published:** 2025-04-25

**Authors:** Sıtkı Ermiş, Uğur Ercan, Aylin Kabaş, Önder Kabaş, Georgiana Moiceanu

**Affiliations:** 1 Department of Horticulture, Faculty of Agriculture, Eskişehir Osmangazi University, Eskişehir 26040, Türkiye; ermis@ogu.edu.tr; 2 Department of Informatics, Akdeniz University, Antalya 07070, Türkiye; 3 Department of Organic Farming, Manavgat Vocational School, Akdeniz University, Antalya 07070, Türkiye; akabas@akdeniz.edu.tr; 4 Department of Machine, Technical Science Vocational School, Akdeniz University, Antalya 07070, Türkiye; okabas@akdeniz.edu.tr; 5 Department of Entrepreneurship and Management, Faculty of Entrepreneurship, Business Engineering and Management, National University of Science and Technology Politehnica Bucharest, 060042 Bucharest, Romania

**Keywords:** ornamental pumpkin, machine learning, seed classification, morphological analysis, artificial intelligence

## Abstract

Ornamental pumpkin (*Cucurbita pepo* L. var. *ovifera*) seeds are highly morphologically variable, and their classification is hence a complex task for the seed industry. Efficient and accurate classification is critical for agricultural production, breeding programs, and seed sorting for commerce. This study employs machine learning models—Random Forest (RF), LightGBM, and k-Nearest Neighbors (KNN)—to classify ornamental pumpkin seeds based on their morphological (mass, elongation, width, thickness) and colorimetric characteristics (L*, a*, b* values from CIELAB color space). Prior to model training, the data set was preprocessed through normalization and balancing to enhance classification performance. In this study, six different types of ornamental pumpkin seeds were used, with a total of 900 (150 each of SDE0619, SDE1020, SDE1620, SDE2621, SDE4521, and SDE7721). The classification performance of the models was evaluated using different metrics, such as Accuracy, Balanced Accuracy, Precision, Recall, F1 Score, Matthews Correlation Coefficient (MCC), and Cohen’s Kappa. Among the tested models, the RF model performed best, with Accuracy of 0.959, Balanced Accuracy of 0.961, Precision (Macro) of 0.962, Recall (Macro) of 0.961, F1 Score (Macro) of 0.961, MCC of 0.951, and Cohen’s Kappa of 0.951. In contrast, the worst classification performance of the tested models was with the KNN model across all the evaluation metrics. These outcomes reflect the potential of machine learning-based approaches for seed classification automation, error minimization in seed classification, and maximization of efficiency in the seed industry. The high classification performance of the Random Forest model with 95.9% accuracy and 0.951 MCC value shows that artificial intelligence-based automatic classification of ornamental pumpkin seeds according to their morphological and colorimetric characteristics can make significant contributions to the seed industry, while the integration of this approach into seed sorting and quality determination processes can enable the creation of effective breeding schemes for optimum seed selection by maximizing the accuracy of agricultural processes.

## 1. Introduction

The Cucurbitaceae family, comprising approximately 130 genera and 960 species, includes economically significant crops such as melon, watermelon, cucumber, and squash [1,2,3]. Among these, *Cucurbita pepo*, *C. moschata*, and *C. maxima* are the most cultivated species globally due to their agricultural value [4,5]. *Cucurbita pepo* L., known as summer squash, is a species native to North America, with wild forms found in northeastern Mexico and southern, southeastern, and central regions of Central America [6,7,8]. *C. pepo* contains two subspecies: *C. pepo* subsp. *pepo* and *C. pepo* subsp. *ovifera* [9]. While subsp. *pepo* is primarily cultivated for food, subsp. *ovifera* includes ornamental types such as acorn, scallop, crookneck, and straight-necked squashes. These display significant variation in fruit shape, size, color, and surface texture, reflecting the morphological richness of ornamental gourds [10]. *C. pepo* is one of the species that shows the most polymorphism in terms of fruit characteristics (variety in size, shape, and color); *pepo* and *ovifera* subspecies can be classified into eight different morphotype groups [11,12]. The subspecies *ovifera* (ssp. *texana*), native to the southeastern and central United States, includes ornamental types such as acorn (*C. pepo* var. *turbinata*), scallop (var. *clypeata*), crookneck (var. *torticollis*), and straight-necked squash (var. *recticollis*), which are cultivated for both ornamental and culinary purposes due to their diverse shapes, textures, and striking color patterns [13,14,15,16]. Ornamental gourds exhibit diverse shapes, textures, and colors, reflecting their broad genetic variability [17,18]. This phenotypic diversity complicates manual classification, making automated approaches like machine learning particularly valuable. In addition to their morphological richness, ornamental pumpkin seeds are nutritionally valuable, containing unsaturated fatty acids and antioxidants [19]. However, nutritional traits were not within the scope of this classification study. Recent morphological analyses of *C. pepo* seeds have underscored the importance of quantitative traits such as length, width, and thickness, as well as derived ratios, in species identification and classification. These parameters are particularly useful in distinguishing the larger seeds of *C. pepo* subsp. *pepo* from the smaller seeds of subsp. *ovifera* [13,20,21].

Recent morphological analyses of C. pepo seeds have underscored the importance of quantitative traits such as length, width, and thickness, as well as derived ratios, in species identification and classification. These parameters are particularly useful in distinguishing the larger seeds of *C. pepo* subsp. pepo from the smaller seeds of subsp. *ovifera* [13,20,21]. The seeds of *C. pepo* var. ovifera show wide morphological variation, including differences in size, shape, coat texture, and color [19], which are critical for effective classification using seed sorting machines [22,23].

However, despite their importance, no previous study has systematically applied machine learning techniques to classify ornamental pumpkin (*C. pepo* var. *ovifera*) seeds using morphometric and colorimetric traits. This study presents a novel integration of AI-driven methods into the classification of morphologically diverse ornamental seeds, addressing a significant gap in seed technology and phenotypic data analysis.

During the determination of these seed traits, such a large amount of information is generated that it is almost impossible for humans to analyze such information quickly and effectively in a laboratory environment [24]. Therefore, erroneous results can cause economic losses for seed companies. By law, seeds to be used in agricultural production must meet certain quality standards, and seed companies must conduct control tests that determine these standards. This can lead to the generation of a large amount of data during agricultural harvest. Based on this demand, seed technology research has focused on determining the aspects associated with the sorting of lots according to the physiological potential of seeds. These measures require a lot of cost, time, and effort. New technologies have been created to solve these problems. Recently developed technologies enable fast and accurate identification and classification of seed traits relevant to quality control. However, pragmatic techniques are required to identify the traits used in the quality assessment of seeds. One of the tools that has attracted the attention of researchers is the use of machine learning and artificial intelligence to sort lots [25,26].

Artificial intelligence and machine learning represent an approach that mimics the human brain and can make decisions by incorporating human characteristics and completing the process in a new way [27]. Machine learning enables computational algorithms to execute targeted operations through the acquisition and interpretation of comprehensive data. The advantage of machine learning lies in its ability to categorize examples efficiently [28]. Machine learning employs multi-layered mathematical operations to learn and process complex data, and it is also designed to imitate the human brain. Classification operations are conducted by processing data through machine learning algorithms [29]. Thus, the analysis of large volumes of data generated during seed quality characterization is facilitated.

Recently, many researchers have conducted various studies using machine learning (ML) techniques to classify various seeds. Studies have examined various algorithms including Support Vector Machines [30,31], decision trees [32], and Convolutional Neural Networks [33] and have shown their effectiveness in classifying seeds based on features such as color, shape, texture, and internal structure. Researchers have used image processing techniques and fuzzy clustered random forests for wheat seed classification and achieved high accuracy [34]. Chen et al. (2021) [31] conducted a study that increased product purity and reduced weed competition by separating weed seeds from crop seeds using machine learning methods, while Thyagharajan and Kiruba Raji (2018) [35] used machine learning algorithms to accurately identify different seed types based on visual features. Huang et al. (2019) [36] used machine vision to classify maize seed defects based on shape and texture and reported that CNNs achieved 95% accuracy in seed defect classification compared to SURF+SVM. Dheer and Singh (2019) [37] analyzed the performance of k-NN, LDA, Logistic Regression (LR), and Naive Bayes (NB) algorithms using 100 datasets to classify seven different wheat varieties; Guevara-Hernández and Gómez Gil (2011) [38] developed a classification model based on LDA and k-NN with 10 wheat and 10 barley images; and [39] determined six physical properties of three pumpkin seed species with BPNN and RBNN (Radial Basis Neural Network) methods and revealed the diversity in this area. Other applications include the identification of low-quality soybean seeds [40] and the classification of Jatropha curcas seeds using radiographic images [41].

Despite the potential of artificial intelligence and machine learning techniques in seed classification, there is a critical lack of research on the application of these methods, especially in morphologically diverse species such as ornamental pumpkins (*Cucurbita pepo* var. *ovifera*). Existing studies generally focus on basic varieties of agricultural crops, but no approach has been presented to systematically analyze complex phenotypic traits (biophysical parameters, colorimetric indices) of ornamental pumpkins, which have both esthetic and economic value, and integrate these traits with machine learning models. In particular, the lack of quantitative mapping of subtle morphometric differences between subspecies leads to a lack of automation in seed quality control and the inability to develop data-based decision-making mechanisms for the conservation of genetic resources. However, as a result of the literature search, no study was found on the classification of ornamental pumpkin (*Cucurbita pepo* L. var. *ovifera* (L.) Alef.) seeds using machine learning models. The innovative contribution of this research comes from the application of machine learning and analytical approaches to discriminate between ornamental gourd kernels with similar physical characteristics.

This study aims to establish a machine learning-driven framework to accurately classify six ornamental pumpkin seed cultivars by integrating morphological properties (mass, elongation, width, thickness) with colorimetric data (CIELAB L*, a*, b* values). We use three robust algorithms selected for their distinct capabilities in processing complex biological datasets: Random Forest (RF), k-Nearest Neighbors (kNN), and LightGBM. By correlating these measurable seed traits with taxonomic identity, our models address a critical gap in *Cucurbita pepo* var. *ovifera* research by enabling the rapid and non-destructive discrimination of subspecies. Beyond taxonomic precision, this study directly supports agricultural biodiversity conservation by providing tools to validate seed cultivars and preserve genetic integrity in germplasm banks. Furthermore, the developed protocol provides scalable solutions for industrial seed sorting systems, reducing reliance on labor-intensive manual screening while improving accuracy in quality control processes.

## 2. Materials and Methods

### 2.1. Plant Material and Experimental Site

This study utilized ornamental gourds collected from five distinct provinces in Turkey: Eskişehir, Balıkesir, Ankara, Manisa, and Bursa. The research was conducted at the “Local Seed Center”, situated in the Odunpazarı district of Eskişehir province, within the geographic coordinates of 39°74′11″–39°74′16″ N latitude and 30°44′87″–30°44′99″ E longitude. The experimental site is positioned at an altitude of 788 m above sea level and exhibits comparable agro-ecological conditions. The experimental study was conducted in Eskişehir province, which is characterized by a temperate continental climate. Climatic conditions during the growing season were moderate to warm, with average temperatures between 18–30 °C and low relative humidity (d Soil samples collected from a depth of 0–30 cm prior to sowing were analyzed in an accredited soil laboratory to assess the physical and chemical properties of the experimental site. According to the results, the soil was classified as clay–loam, with 58% water saturation, 0.646 dS/m electrical conductivity, and a slightly alkaline pH of 8.08. The organic matter content was 2.08%, and lime content was 6.71%. Available phosphorus and potassium were 65.27 and 258.5 kg/da, respectively. Micronutrient concentrations included 3.0 mg/kg Zn, 2.9 mg/kg Fe, 1.3 mg/kg Mn, and 3.6 mg/kg Cu, indicating a nutrient-rich profile conducive to stable seed development.

A Randomized Complete Block Design (RCBD) was employed for this study. The experimental arrangement included two replications, with all genotypes randomly assigned within each block. Sowing was conducted with a row spacing of 1.5 m and an in-row spacing of 0.8 m, ensuring that each plot contained a single genotype. Within each plot, a single row consisting of ten plants was established. Harvesting occurred when the leaves had dried and the fruit peduncle had desiccated to a point allowing detachment from the plant stem. At this stage, fruits that had undergone self-pollination with their own pollen were meticulously labeled and harvested. The collected fruits were then halved, and the seeds within were carefully extracted. Subsequently, these seeds were transferred to a controlled drying chamber maintained at 25 °C with 35% relative humidity for 72 h, to serve as seed stock for the following planting season.

Seed morphometric parameters (elongation, width, thickness) were quantified using ImageJ v1.53k (National Institutes of Health, Bethesda, MD, USA) with the following protocol: (1) grayscale conversion and scale calibration using a 1 mm reference object included in all images; (2) elongation measurement via the Feret diameter ratio (maximum/minimum) using the ‘Straight Line’ tool; (3) width and thickness determination through orthogonal axis analysis with the ‘Oval Selection’ tool, applying a fixed threshold of 80–255 to exclude background noise. The ‘Analyze Particles’ function was configured to detect particles >0.5 mm^2^ with circularity 0.6–1.0 to ensure accurate seed boundary identification [42].

**Figure 1:**
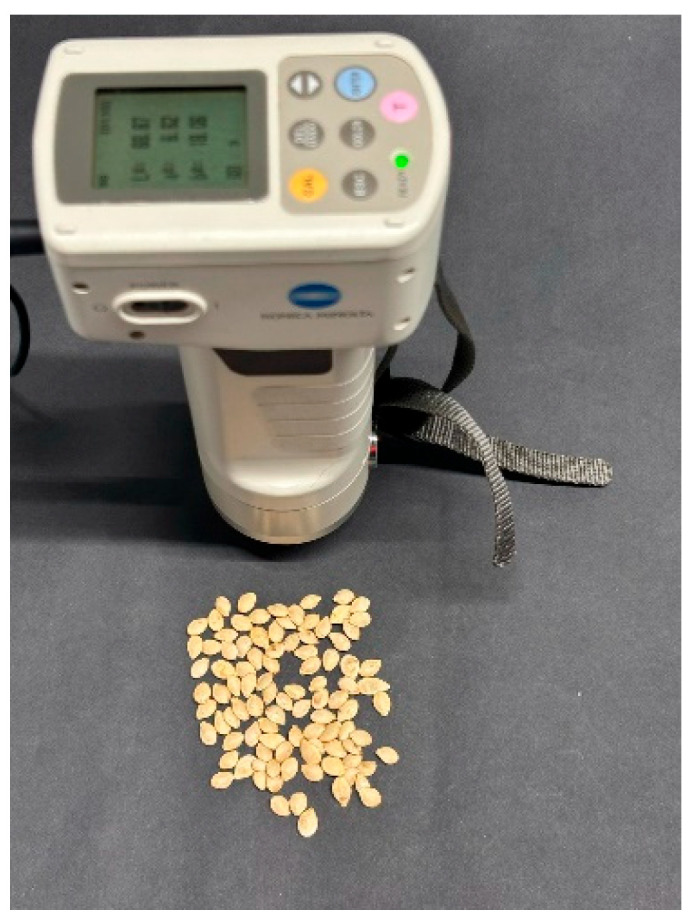
The color measurement of ornamental pumpkin seeds was conducted utilizing a colorimeter.

The visible color properties (L, a, b) of ornamental pumpkin seed varieties were determined by using a digital colorimeter (Chroma Meter CR-400 (Konica Minolta, Tokyo, Japan) (Figure 1).

The mass of ornamental pumpkin seeds (W, in grams) was quantified using a high-precision analytical balance (Model GX-4000, A&D Company, Ltd., Tokyo, Japan) capable of measurements accurate to ±0.01 g.

**Figure 2:**
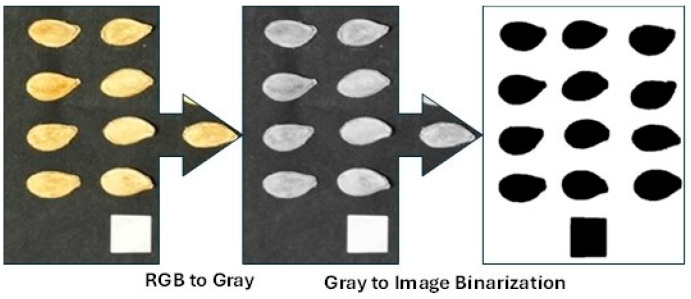
Processing stages of images for the ImageJ program.

The images of ornamental pumpkin seeds were photographed in a special box that did not allow external light to enter and prevented shadowing. The lighting system and camera were arranged with the camera securely mounted perpendicular (90°) to the box surface. The seeds were set against a dark backdrop to make image processing easier. Photographs of 150 randomly selected seeds from each variety were taken for image analysis. The images of the captured seeds were processed using the open-source software ImageJ v1.53k. The main photographs were first converted to gray-scale and then to binary (black and white) format, as in Figure 2.

**Figure 3:**
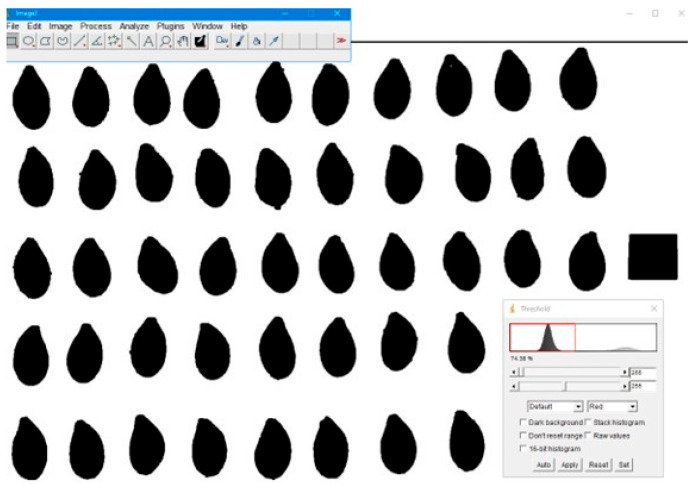
Determination of linear dimensions with ImageJ program.

Threshold values were identified using the Otsu algorithm [43]. The width, length, and thickness values were measured from the converted images according to the shape in which the reference measurement was taken (Figure 3).

### 2.2. Machine Learning

**Figure 4:**
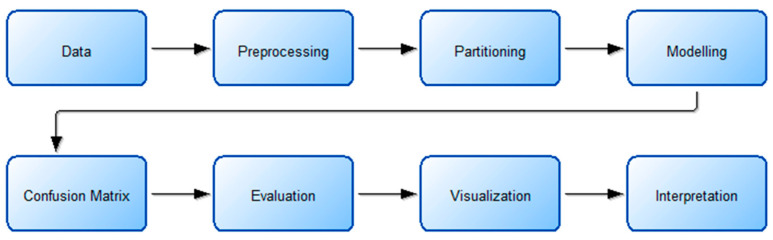
Working flow chart.

**Figure 5:**
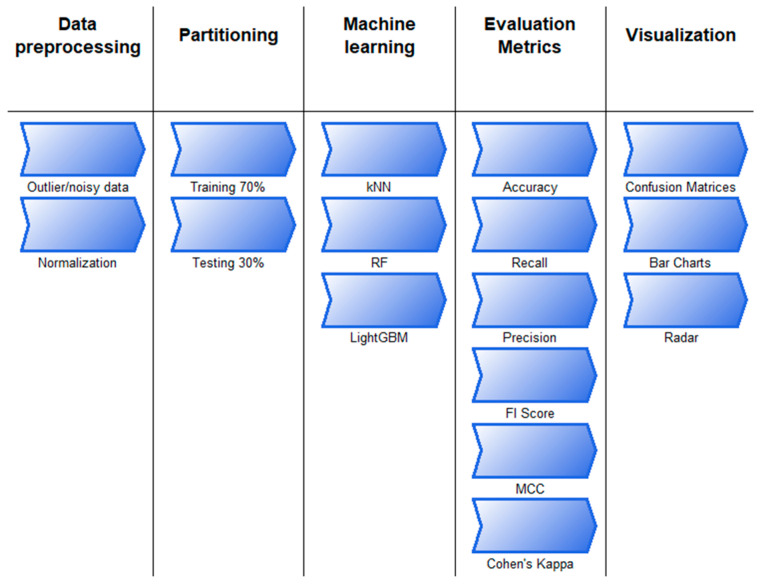
Stages and details on the way from data to information.

The discovery of knowledge from data is defined as a process in computer and data science and is called data mining. In fact, it is the process of revealing the hidden information in the data so that it can be understood [44,45]. This process consists of successive steps. The machine learning or modeling step is one of the steps in this process, and kNN, LightGBM, RF, or other known methods are applied in this step. The flow chart describing the process undertaken during the knowledge discovery step and the details of the actions performed in these steps are shown in Figure 4 and Figure 5.

The study begins by selecting a suitable data set based on the problem’s objective. Next, the data undergoes preprocessing, during which missing, noisy, or outlier observations are identified. In this step, min–max normalization is also applied. The data set is then split into training and testing sets, with the training data being balanced to enhance model performance. The following step involves modeling, where machine learning algorithms are applied. The test data set is subsequently used to assess the trained model. The process then includes the following steps: obtaining the confusion matrix, obtaining the assessment metrics, visualization, and interpretation.

This study aims to classify ornamental pumpkin seeds by means of machine learning methods using morphological, physical, and color characteristics.

For the classification process, Random Forest, k-Nearest Neighbors, and LightGBM techniques were utilized. In most classification studies in agriculture, commonly used machine learning methods are LightGBM, RF, and k-NN algorithms [46,47,48].

#### 2.2.1. Random Forests

**Figure 6:**
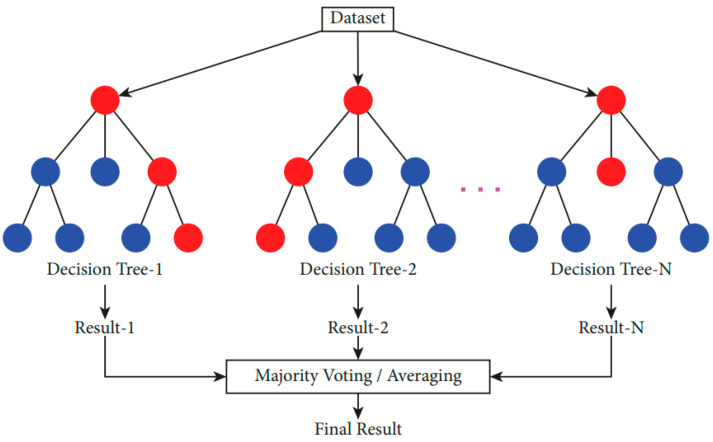
Illustration of the RF classification [54].

Random Forests (RFs) consist of multiple Decision Trees that are randomly generated from a data set [49,50]. This approach involves the construction of several individual, unpruned decision trees by introducing randomness in the splitting process at each node. The main goal here is to improve accuracy by combining several approximate trees into an ensemble. This is frequently more effective than a single tree with exact divisions [51]. It is a useful method for processing data sets that contain missing or outlier information and for completing transactions in big data sets rapidly. The result of each decision tree in the forest is used to determine the class. The class with the most votes indicates the result of the RF model [52,53]. Figure 6 shows how the RF classification works.

#### 2.2.2. LightGBM

LightGBM, a high-performance machine learning algorithm that aims to improve the efficiency of gradient-boosting decision tree (GBDT) algorithms, offers faster training times and lower memory usage compared to traditional GBDT methods. The algorithm uses a leaf-oriented tree growth strategy. Thus, at each step, it expands the leaf that provides the highest error reduction. This approach increases the accuracy of the model while reducing the overfitting [55,56]. The LightGBM algorithm proposes two new features: Gradient-Based One-Side Sampling (GOSS) and Exclusive Feature Bundling (EFB). With GOSS, instead of using the entire data set, it uses the subsampled data set produced from the data. EFB reduces the processing complexity by converting sparse features into more frequent features [57,58].

#### 2.2.3. k-Nearest Neighbors (kNN)

The KNN algorithm, which is a controlled machine learning method, is simple, understandable, scalable, and also robust against noisy data. These advantages make it a strong opponent against other classification algorithms [59,60]. It is a similarity-based classification method and uses the nearest K neighbor in the training data to determine the class of a new data point. When a new data point is given, the distance between all examples in the training set is calculated. Once the distances between all data points have been calculated, the K neighbor with the smallest distance is determined. The majority class of the selected K neighbor is determined and the label of the new data point is assigned [61].

The tuning parameters for the RF, kNN, and LightGBM models that yielded the best performance are presented in Table 1.

## 3. Results and Discussion

### 3.1. Statistical Analysis

Table 2 displays the findings of the descriptive statistical analysis performed on the study’s data. Eskişehir Osmangazi University’s Faculty of Agriculture collected the study’s data. Variety is the target variable. It is categorical and takes six different values. This variant takes the values SDE0619, SDE1020, SDE1620, SDE2621, SDE4521, and SDE7721. There are 150 samples of all types in equal frequencies. ML modeling, descriptive statistics, and all graphics were performed using Python (3.13.3) programming language.

### 3.2. Evaluation Metrics

**Figure 7:**
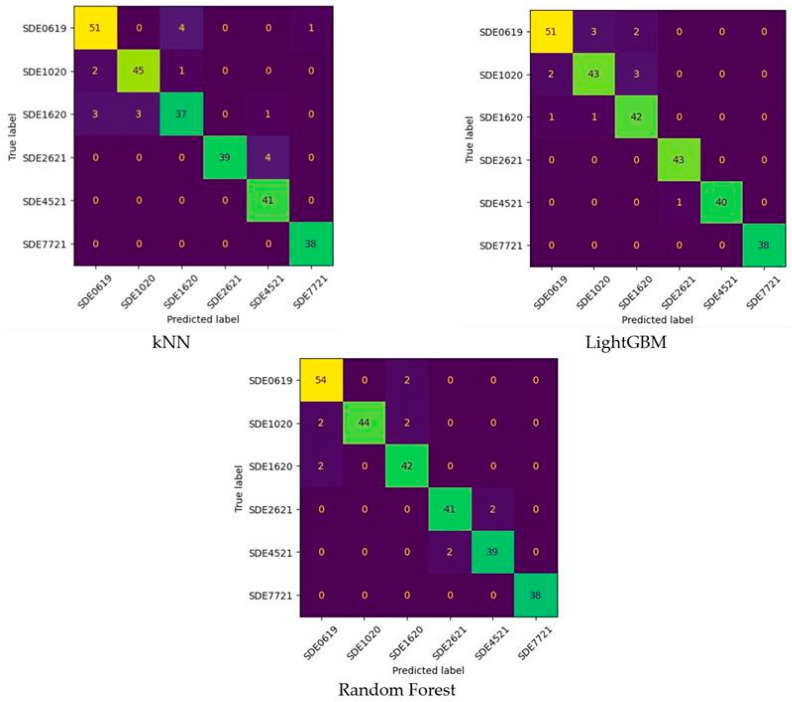
Confusion matrices of machine learning models.

The accuracy metric in classification analysis determines a classification’s overall success [62]. For the evaluation of problems with more than two classes, the metrics of Accuracy, Weighted Accuracy, Precision (macro), Recall (macro), Matthew’s Correlation Coefficient, Cohen’s Kappa and F1-Score (macro) are used [63,64]. In this study, a total of three models were established. The testing partition confusion matrices and results for all models are shown in Table 3 and Figure 7 respectively.

When the Confusion Matrices were interpreted, as shown in Figure 7, the following results were found. In the classification of SDE0619 species, kNN, LightGBM, and RF models misclassified five, five, and two seeds, respectively. The most successful model for SDE0619 type was the RF model. In the classification of SDE1020 species, kNN, LightGBM, and RF models misclassified three, five, and four seeds, respectively. The most successful model for SDE1020 type was the kNN model. In the classification of SDE1620 species, kNN, LightGBM, and RF models misclassified seven, two, and two seeds, respectively. The most successful models for SDE1620 type were the LightGBM and RF models. In the SDE2621 type, the LightGBM model produced a perfect classification. On the other hand, the RF model had two and the KNN model had four wrong classifications. In the SDE4521 type, the kNN model made a perfect classification. On the other hand, the RF model had two and the LightGBM model had four wrong classifications. In the SDE7721 type, all ML models made perfect classifications.

As shown in Table 3, the following findings were acquired when interpretations were based on machine learning techniques and metrics.

### 3.3. Based on Machine Learning Method

When we interpreted the methods, it could be seen that the most successful model was the model established by the RF method, while the second most successful model was the model established by the LightGBM method; the model established by the kNN method was the most unsuccessful model. In fact, although the LightGBM model’s results and the RF model’s results were very close, more successful results were obtained in the model established by the RF method.

### 3.4. Based on Evaluation Metrics

Since it gauges the general success of ML models, the accuracy metric is significant. It is calculated by dividing the number of accurate predictions by the total number of forecasts. The results of RF, LightGBM, and kNN models according to accuracy metrics are 0.96, 0.95, and 0.93, respectively. According to these results, all models had high general classification achievements. However, the model installed with RF classified the ornamental pumpkin seeds with 96% accuracy.

In balanced accuracy, a value is obtained by dividing the number of objects correctly classified in each class by the total number of objects. Then, the sum of the values obtained is divided by the total number of classes. According to the Balanced Accuracy Metric, the performance of all models varies between approximately 93% and 96%. The RF model has the best Balanced Accuracy value, and the success of this model is 96.1%. The Accuracy and Balanced Accuracy values are very close. The reason for this is that the data set is balanced and that the models predict the classes well.

Since the Recall Macro Metric and the Balanced Accuracy Metric are computed using the same formula, their results are the same. Recall measures the proportion of actual positive samples that are correctly identified by the model. In the Recall Macro metric, each class is of equal importance. All classes are balanced in the data set in our study. Since class-based recall values are also high, the Recall Macro achieves a high value. The model developed using the RF technique yields the best Recall Macro value, which is roughly 0.96. Values close to 1 in this metric indicates that the model accurately estimates the real positives.

The Precision Macro metric can be defined as “how much of what the model positive predicts is actually true”. It is determined by computing the arithmetic average of the Precision scores obtained for each class. In this metric, all classes are of equal importance. The data set is balanced in our study. Since class-based precision values were also high, the Precision Macro achieved a high value. Based on the Precision Macro metric, the performance of all models ranged from around 0.93 to 0.96. The highest Precision Macro score was approximately 0.96, achieved by the model built using the RF method.

The F1 Score Macro metric is obtained by calculating the harmonic mean of the macro recall and macro precision values. In this metric, high frequency and low frequency classes have the same effect. While the metric receives a value between [−1, +1], values close to 1 are a sign of a good performance in all classes. Based on the F1 Score Macro metric, all models exhibit performance ranging from approximately 0.93 to 0.96. The highest F1 Score Macro value is around 0.96, achieved by the model developed using the RF method.

**Figure 8:**
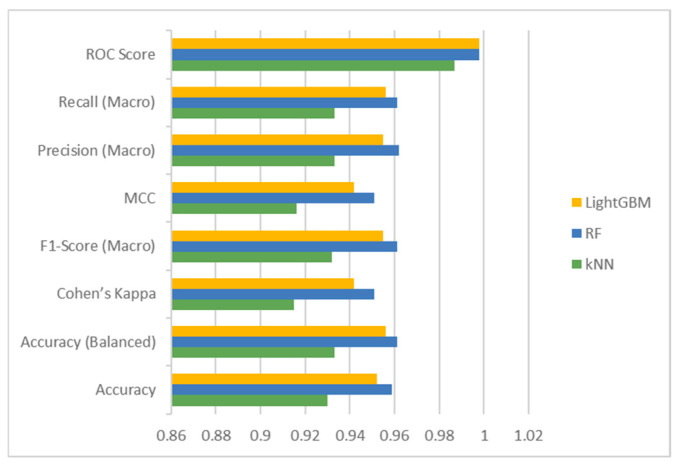
Results of LightGBM, RF, and kNN models.

**Figure 9:**
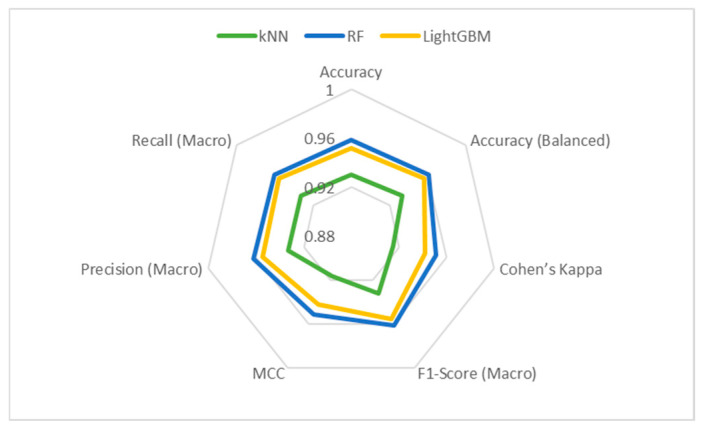
Radar graph of LightGBM, RF, and kNN models.

Figure 8 and Figure 9 display the Bar and Radar graphs of the ML models. In the Bar graph, where the metric results are presented visually, it can be seen that the RF model outperforms the other models. In the Radar graph, the RF model can be considered more successful than the other models because it presents a more symmetrical image. In this type of graph, it is also possible to compare in which metrics the models are better or weaker.

MCC is a robust metric for evaluating model performance. It takes [−1, +1], and reflects the accuracy and reliability of the model’s predictions [65,66]. If the MCC value is close to 1, the model’s predictions are good. According to the MCC metric, the performance of all models varies between approximately 0.92 and 0.95. The RF model has the best MCC value, and this is 0.951. The number of accurately classified elements has a great impact on the MCC metric. Therefore, an error in one of the classes will severely reduce the outcome of the metric. Classification errors made by the kNN model (SDE1620:7 errors, SDE2621:4 errors) have caused MCC metric result to have a lower value than others.

Cohen’s Kappa or Kappa is used to assess the success of a classifier. An excellent classification takes a value of 1, while an estimate of the model that is completely independent without real classification takes a value of 0. When there is no harmony between the estimation of the model and the real value, the Kappa receives a negative value [63,67]. According to the Kappa metric, the performance of all models varies between approximately 0.92 and 0.95. The RF model has the best Kappa value, and this is 0.951. In this study, the incorrect classification of some observations of the SDE1620 and SDE2621 types caused the Kappa value of the kNN model to receive a low value compared to other model results.

As mentioned before, there have been studies on the classification of fruits and vegetables using various features. Ercan et al. [68] achieved the best results using the model established with RF methods. The Accuracy, Accuracy (Weighted), Precision (Macro), Recall (Macro), F1 (Macro), MCC, and Cohen’s Kappa results were 0.9866, 0.9891, 0.9823, 0.9891, 0.9870, 0.9817, and 0.9809, respectively. Koklu et al. [47] achieved the best Accuracy (0.886), Precision (0.928), Specification (0.915), and F1-Score (0.895) results with the SVM method in the classification of ornamental pumpkin seeds, while the best Recall (0.865) result was achieved using the ANN method. Çetin et al. [23] achieved the best Accuracy (0.845), Precision (0.848), True Positive Rate (0.840), and F1-Score (0.844) results with the ANN method in the classification of ornamental pumpkin seeds. In another part of their studies, they achieved the best Accuracy (0.855), Precision (0.768), True Positive Rate (0.730), and F1-Score (0.749) results with the RF method. Li et al. [69] classified ornamental pumpkin seeds with 95.20% accuracy in their study. Gulzar et al. [33] successfully classified various seeds with 99% accuracy, 0.99 Recall, and 0.99 Precision in their study. In this study, although the results were close to each other in all metrics, the RF model was the most successful model. The Accuracy, Accuracy (Balanced), Precision (Macro), Recall (Macro), F1 Score (Macro), MCC, and Cohen’s Kappa results were 0.959, 0.961, 0.962, 0.961, 0.961, 0.951, and 0.951, respectively. As can be seen from the metric results and Confusion Matrices, the ML models were successful and the ornamental pumpkin seeds were successfully classified.

## 4. Conclusions

One of the major challenges in the vegetable seed industry involves not only removing foreign materials from seed batches but also accurately distinguishing different varieties belonging to the same species. This issue is particularly acute in ornamental pumpkin seeds, where traditional classification systems—such as optical sorting and binocular microscopy—are limited in their precision due to morphological similarities. While these conventional approaches often achieve classification accuracies of around 70–80%, our machine learning-based method using Random Forest achieved a precision of 96.2%, highlighting a significant improvement over existing systems.

The aim of this study was to classify ornamental pumpkin seed genotypes using morphometric and colorimetric features by evaluating the performance of Random Forest (RF), LightGBM, and k-Nearest Neighbors (kNN) algorithms. The RF model outperformed the others in all evaluation metrics, achieving Accuracy, Precision, Recall, F1 Score, MCC, and Cohen’s Kappa values exceeding 95%. These results demonstrate the capability of machine learning to robustly discriminate between visually similar seed types in a non-destructive manner.

Despite the strong potential, the implementation of machine learning systems in real-world seed production workflows may require investment in specialized imaging equipment, operator training, and integration with existing seed sorting infrastructure. These practical considerations must be addressed in order to translate research findings into scalable commercial solutions.

In future studies, incorporating larger datasets, real-time image acquisition, and hybrid classification models may further enhance prediction accuracy and operational efficiency. Overall, this research supports both quality control in industrial seed sorting and genetic resource conservation through precise, automated seed classification systems. While traditional methods such as optical sorting and binocular microscopy typically achieve classification accuracies between 70 and 80%, the Random Forest model in this study achieved 95.9% accuracy and 0.951 MCC, demonstrating a substantial performance improvement. The integration of these approaches into commercial pipelines may pave the way for smarter, data-driven seed technologies that support both industrial innovation and genetic diversity preservation.

## Figures and Tables

**Table 1:** Tuning parameters for the RF, kNN, and LightGBM models.

RF	kNN	LightGBM
n_estimators = 200, criterion = ‘entropy’, max_depth = 9, min_samples_split = 2, min_samples_leaf = 2, ma_features = ‘sqrt’	n_neighbors = 5, weights = ‘uniform’, algorithm= ‘kd_tree’, leaf_size = 30, p = 2, metric =‘cityblock’	boosting_type = ‘dart’, num_leaves = 800, max_depth= 7, learning_rate = 0.2, n_estimators = 1500, subsample_for_bin = 350,000

**Table 2:** Descriptive statistics of the data.

	Elongation (mm)	Width (mm)	Thickness (mm)	Weight (g)	L	a	b
Min	1.96	1.85	1.27	0.0248	7.75	3.04	1.18
Max	70.34	9.95	20.7	16.71	85.05	66.07	21.17
Std Dev	3.40	1.34	0.76	0.56	6.22	2.17	4.69
Ave	11.86	7.23	2.23	0.10	77.33	5.33	11.96
Skewness	5.84	0.08	17.58	29.66	−9.04	24.63	−0.28
Curtosis	96.50	−1.18	399.43	886.31	98.09	687.71	−0.97
Number of Observations: 900							

**Table 3:** Results of metrics for testing partition.

Metrics	kNN	RF	LightGBM
Accuracy	0.930	0.959	0.952
Accuracy (Balanced)	0.933	0.961	0.956
Cohen’s Kappa	0.915	0.951	0.942
F1-Score (Macro)	0.932	0.961	0.955
Matthews Correlation Coefficient	0.916	0.951	0.942
Precision (Macro)	0.933	0.962	0.955
Recall (Macro)	0.933	0.961	0.956

## Data Availability

The raw data supporting the conclusions of this article will be made available by the authors on request.
